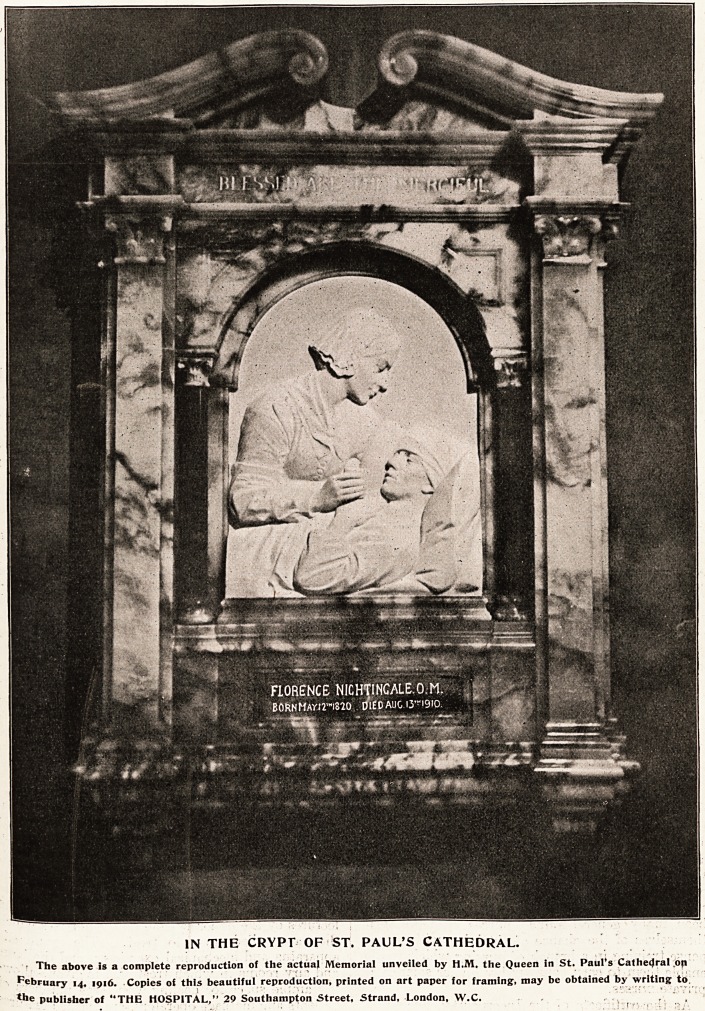# The Florence Nightingale Memorial

**Published:** 1916-03-04

**Authors:** 


### THE FLORENCE NIGHTINGALE MEMORIAL.

### IN THE CRYPT OF ST. PAUL'S CATHEDRAL.

**The above is a complete reproduction of the actual Memorial unveiled by H.M. the Queen in St. Paul's Cathedral on February 14, 1916. Copies of this beautiful reproduction, printed on art paper for framing, may be obtained by writing to the publisher of "THE HOSPITAL," 29 Southampton Street, Strand, London, W.C.**

## Figures and Tables

**Figure f1:**